# Composite barrier membrane for bone regeneration: advancing biomaterial strategies in defect repair†

**DOI:** 10.1039/d4ra07623k

**Published:** 2025-01-15

**Authors:** Qingbin Han, Delu Zhao, Xiaohong Wang, Mengyao Shang, Wenbin Zhou, Qing Li, Hui Song

**Affiliations:** a School and Hospital of Stomatology, Cheeloo College of Medicine, Shandong University, Shandong Key Laboratory of Oral Tissue Regeneration, Shandong Engineering Research Center of Dental Materials and Oral Tissue Regeneratioon, Shandong Provincial Clinical Research Center for Oral Diseases Ji'nan 250012 China lq580202@sdu.edu.cn Songhui@sdu.edu.cn; b Department of Oral and Maxillofacial Surgery, Linyi People's Hospital Lin'yi 276000 China

## Abstract

Bone defects represent a significant challenge in clinical practice, driving the need for innovative solutions that effectively support bone regeneration. Barrier membranes, due to playing a critical role in creating an environment conducive to bone regeneration by preventing the infiltration of non-osteogenic tissues, are widely applied to bone repair. However, inadequate spatial stability and osteogenesis-promoting ability often limit current barrier membranes. In response to these challenges, we have developed an advanced gelatin methacrylate/hydroxyapatite/hydroxyapatite membrane (GelMA/HAp/HAM) composite biomaterial designed as a barrier membrane with superior spatial stability and optimal degradation properties. The GelMA/HAp/HAM composite features a bilayer structure, with each layer possessing distinct properties: the dense hydroxyapatite membrane (HAM) acts as a barrier to prevent connective tissue infiltration. In contrast, the porous gelatin methacrylate/hydroxyapatite (GelMA/HAp) hydrogel layer promotes osteogenesis. Studies have demonstrated the composite's excellent biocompatibility and its significant osteogenic differentiation enhancement. This composite membrane holds great promise for clinical applications in bone defect repair, providing a new avenue for improving patient outcomes in regenerative medicine.

## Abstract 2

Preparation of GelMA/HAp/HAM barrier membranes. Bone defect regeneration of rat calvaria *in vivo*.

## Graphical abstract

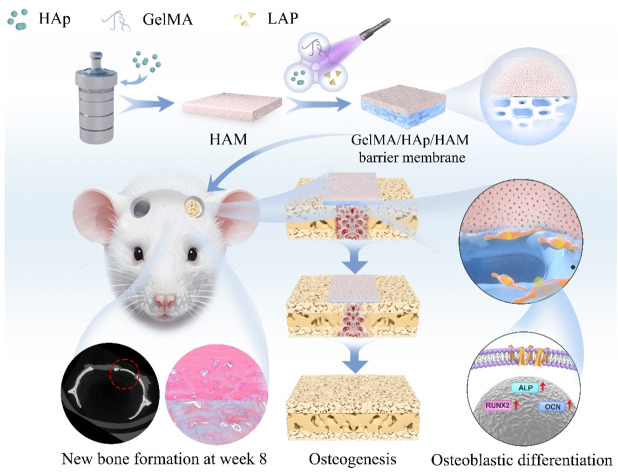

## 1. Introduction

Bone defects are common in the maxillofacial region and pose significant therapeutic challenges.^1–3^ Guided bone regeneration (GBR) is a technique designed to augment bone volume based on the characteristics and dimensions of the defect site and has been widely used in bone augmentation.^4–7^ Despite significant advances in surgical treatment, effective reconstruction of sufficient bone volume, especially vertical height, remains a formidable challenge. Currently, GBR techniques are used in clinical practice to treat maxillary bone defects, focusing on restoring alveolar bone thickness and ridge height.^8,9^ Maintaining spatial stability through barrier membranes is critical to successful bone reconstruction for extensive bone defects.^10,11^ However, an ideal material that provides safety and efficacy in barrier function remains elusive.

Commonly used resorbable collagen barrier membranes in clinical practice are rapidly resorbed but have poor spatial stability.^12–14^ Non-resorbable polytetrafluoroethylene (PTFE) membranes, on the other hand, are prone to postoperative soft tissue dehiscence and often require secondary surgery for removal, increasing trauma.^5,15^ Although titanium meshes offer favorable mechanical properties and maintain relative spatial stability, their morphology differs significantly from that of alveolar bone,^16^ leading to a higher incidence of exposure and often requiring removal.^17–19^ Therefore, there is an urgent need to develop a biomaterial that combines high bioactivity, safety (to prevent soft tissue dehiscence), and robust spatial stability for use in GBR techniques to repair maxillofacial bone tissue defects.

In the field of bone tissue engineering, gelatin methacrylate (GelMA), a versatile scaffold material in bone tissue engineering, has gained attention for its excellent inherent bioactivity, biocompatibility and biodegradability,^20–22^ promoting cell adhesion and proliferation.^23,24^ Its porous structure facilitates nutrient exchange, supporting cellular activities. Hydroxyapatite (HAp), with its osteoconductive properties and chemical similarity to native bone,^25^ has demonstrated the potential to enhance bone regeneration. Hap is stable, non-toxic, and elicits minimal inflammatory responses, making it an ideal candidate for integration into scaffold materials.^26–29^ Research suggests that achieving optimal spatial stability and a controlled degradation rate are key to developing effective GBR materials.Hydroxyapatite membranes (HAM) may address these requirements with their mechanical strength and tunable degradation rates.

We hypothesize that a bilayer composite barrier membrane combining HAp and GelMA will provide enhanced spatial stability, support sustained calcium ion release, and promote osteogenic differentiation, addressing the limitations of current GBR materials. The proposed bilayer membrane features a dense HAM as the outer layer to act as a physical barrier against connective tissue invasion and a porous GelMA/HAp hydrogel as the inner layer to facilitate osteogenesis. The dense HAM, synthesized under high-temperature and high-pressure conditions, exhibits excellent mechanical strength, hydrophobicity, and controlled degradation, providing a stable framework for osteogenesis. In contrast, the porous GelMA/HAp hydrogel enhances bioactivity and supports bone regeneration.

**Scheme 1:**
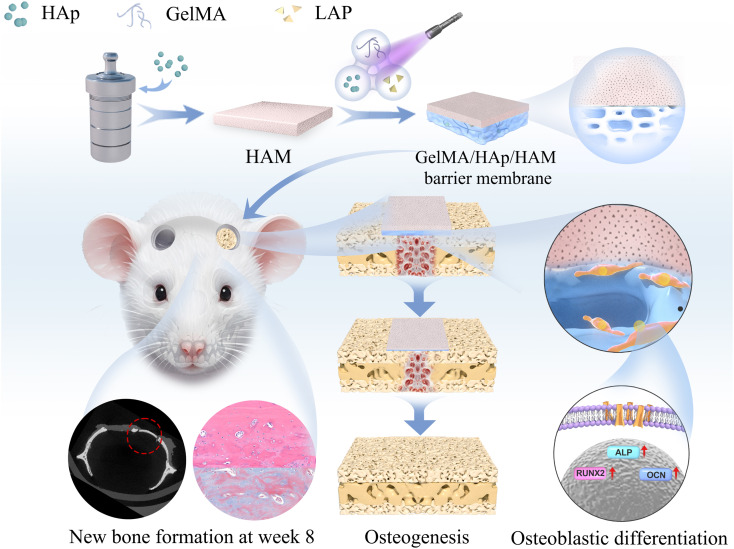
Preparation of GelMA/HAp/HAM barrier membranes. Bone defect regeneration schematic of rat calvaria *in vivo*.

We conducted a series of *in vitro* and *in vivo* experiments to test this hypothesis and evaluate the composite membrane's mechanical properties, degradation profile, biocompatibility, and osteogenic potential. This study demonstrates the membrane's capability to support new bone growth by offering a stable, bioactive, and degradable platform, highlighting its potential for application in maxillofacial bone defect repair (Scheme 1).

## 2. Materials and methods

### 2.1 Materials

HAp microspheres, lithium phenyl-2,4,6-trimethylbenzoylphosphinate (LAP), penicillin (PG) were purchased from Sigma-Aldrich (Sigma-Aldrich, St. Louis, MO, USA). Cell Counting Kit-8 (CCK8), β-glycerophosphate (β-GP), ascorbate acid (Vc) and dexamethasone (DXMS) were purchased from Beyotime (Beyotime, Shanghai, China). Alpha modified eagle medium (α-MEM), fetal bovine serum (FBS), streptomycin (SM) were purchased from Thermo Fisher (Thermo Fisher Scientific, Waltham, MA, USA). Gelatin methacrylate (GelMA) were purchased from EFL (Engineering for life, Suzhou, China). Phosphate buffer saline (PBS) were purchased from Servicebio (Servicebio, Wuhan, China). Ca(NO_3_)_2_·4H_2_O, (NH_4_)_2_HPO_4_ and hexadecyltrimethylammonium bromide (CTAB) were purchased from SCRC (Sinopagic Chemical Reagents Co., Ltd., Beijing, China). The animals used in this study were purchased from Vital River (Beijing Vital River Laboratory Animal Technology Co., Ltd., Beijing, China) and Use Committee of Shandong University. Animal experiments were conducted following the Ethics and Welfare guidelines of Laboratory Animals. Unless specific otherwise, all reagents were analytically pure and used as is.

### 2.2 Isolation and culture of periodontal ligament stem cells

The tissue block method was used to isolate and culture periodontal ligament stem cells (PDLSCs) from bicuspid or third molars of healthy young (13–25 years old) people. The surface markers of periodontal stem cells (CD29/CD45/CD90/CD105) were detected by flow cytometry, and osteogenic and lipogenic differentiation experiments detected the multidirectional differentiation potential of periodontal stem cells. PDLSCs from the third to fifth generations were employed in all experiments.

### 2.3 Preparation of GelMA/HAp/HAM

2 mmol calcium nitrate Ca(NO_3_)_2_·4H_2_O, 0.2 g hexadecyltrimethylammonium bromide (CTAB), and a certain amount of HNO_3_ were adjusted to pH = 7.0 and dissolved in 20 mL deionized water to form solution 1. Then, 1.2 mmol (NH_4_)_2_HPO_4_ was added to 15 mL H_2_O to form solution 2. After vigorous stirring for 30 min, solution 2 was added to solution 1. After stirring for another 20 min, the mixed solution was transferred to a PTFE bottle in a stainless steel autoclave and maintained at 180 °C for 24 h. Naturally, it is cooled to room temperature and centrifuged to obtain a white residue. The residue was washed once with deionized water, twice with anhydrous ethanol, and dried for 12 h. The filtered and cleaned HAp was formed into a paper-like membrane using a vacuum filtration device. After thorough drying, it was compacted using a tablet press to obtain the final HAM.^30^

GelMA (5 W/V%) and LAP (0.25 W/V%) were dissolved in phosphate buffer saline (PBS) and filtered with a 0.22 μm sterile filter to prepare a gel solution. Hydroxyapatite (3 W/V%) particles were added to form a porous hydrogel layer under ultraviolet light. HAM acts as a barrier, covering the surface of the hydrogel layer. GelMA, HAp, and GelMA/HAp were used as control groups.

### 2.4 Morphology and microstructure

The morphology of the biomimetic bone was characterized using scanning electron microscopy (SEM). X-ray diffraction (XRD, Rigaku, D/MAX-2500/PC) was employed to analyze the phase composition of the membrane. GelMA/HAp and GelMA/HAp/HAM samples, each with identical dimensions of 1 × 1 × 0.2 cm, were gradually heated to 600–800 °C in a high-temperature furnace to completely remove the GelMA matrix and HAM components, leaving only the hydroxyapatite (HAp) residue. Three samples from each group were tested to ensure the accuracy and reliability of the measurements.

The mass of the sample before and after combustion is recorded, and the proportion of residue is calculated, which in turn reflects the relative level of HA content. The surface hydrophilicity of HAM and GelMA/HAp was evaluated by measuring the contact angle (CA) with a drop-shape analyzer.

The mechanical properties, including tensile strength, elastic modulus, and strain at failure, were measured using a universal testing instrument (SUST, China) at a crosshead speed of 1 mm min^−1^. Three samples were tested for each group to ensure accuracy and reproducibility of the results.

### 2.5 Swelling behaviour

The prepared hydrogel was freeze-dried, and then the weight of the freeze-dried gel was recorded as W0. Subsequently, the freeze-dried hydrogel was immersed in PBS (pH = 7.2), and then the sample was filtered with filter paper at 10, 20, 30, 60, 90, and 120 min. The weight of the hydrogel at each time was recorded as *W*_*t*_. Three parallel samples were used for each measurement and calculated using the following formula:SR = (*W*_*t*_ − *W*_0_)/*W*_0_ × 100%

### 2.6 Degradation performance

All samples were freeze-dried to obtain the initial weight *W*_i_ and then immersed in PBS for 24 h. 2.5 U ml^−1^ collagenase was shaken in a constant temperature shaker at 37 °C and 70 rpm min^−1^. The collagenase was replaced every 24 h, and the samples were freeze-dried and weighed to obtain the mass *W*_r_ and record it. The remaining mass percentage after enzyme degradation is calculated as follows:*Q*_d_= (*W*_r_/*W*_i_) × 100%

### 2.7 Cell cytotoxicity assay and proliferation assay

Membranes was prepared in 24-well plates. 1 ml α-MEM medium containing 10% FBS was added and incubated in a 37 °C incubator with 5% CO_2_ for 24 h. Third-generation PDLSCs were seeded into 96-well plates at a density of 5000 cells per well. After 24 h, the α-MEM medium containing 10% fetal bovine serum was removed, and 100 μL per well was added to the 96-well plate containing the stem cells. The plates were placed in an incubator at 37 °C containing 5% CO_2_ for 1 day, 3 days, 5 days. After incubation, CCK-8 assays were performed.

To evaluate the viability of PDLSCs on the membranes, the membranes were prepared in 24-well plates according to the described experimental methods. Third-generation PDLSCs were digested with 1% trypsin, centrifuged at 1000 rpm for 5 min, and suspended in α-MEM containing 10% fetal bovine serum. Cell counting was then performed. Then, the PDLSCs were added to the membranes with 20 000 cells per well and placed in an incubator containing 5% CO_2_ at 37 °C for 72 h. The live-dead assay Calcein AM/ethidium bromide homodimer-1, (Invitrogen, CA, USA) was used to observe the cell conditions under fluorescence microscopy.

### 2.8 Osteogenic differentiation assay

PDLSCs were cultured in an osteogenic medium containing α-MEM, 10% fetal bovine serum, 1% penicillin, 1% streptomycin, dexamethasone (0.1 mM), glycerophosphate (10 mM), and ascorbic acid (50 mg mL^−1^). 7 days after osteogenic induction, samples were rinsed with PBS and subsequently fixed with 4% paraformaldehyde (PFA) for 10 min, then stained using a BCIP/NBT Alkaline Phosphatase Color Development Kit (Beyotime) in the dark. After 5 min, the chromogenic reaction was halted by washing with deionized water. The staining results were then captured using a scanner (Microtek, Shanghai, China). The total ribonucleic acid (RNA) was extracted from the stem cells using trizol reagent (Invitrogen) at 3, 7, and 14 days. The 2-ΔΔCt relative gene expression calculation method was used, and the standardized Ct housekeeping gene glyceraldehyde 3-phosphate dehydrogenase (GAPDH) was used. Reverse transcription-polymerase chain reaction (RT-PCR) detected the expression levels of alkaline phosphatase (ALP), runt-related transcription factor-2 (Runx2), and osteocalcin (OCN). Pure human periodontal ligament stem cell osteoblasts served as the standard control.

### 2.9 Animal experiment

SD rats (male, 8 weeks old, 230 ± 20 g) were raised under laboratory conditions. After 1 weeks of adaptive feeding, surgery was performed in the animal operating room. After sodium pentobarbital anesthesia, the skin over the skull was incised to expose the skull surface. A 5 mm bone trephine was used to prepare circular defects on both sides of the skull to the periosteum. Different materials were implanted on the left side, and the defect on the right side was a blank control. The rats recovered well after the operation and were given 40 000 IU/100 g penicillin to prevent infection for 3 days. The animals were euthanized by carbon dioxide asphyxiation 8 weeks after the operation to obtain samples.

### 2.10 Microcomputed tomography analysis

The skulls of rats from all groups were extracted 8 weeks after surgery, and a microcomputed tomography (micro-CT) scan was performed (Sky Scan 1176, Kontich, Belgium). The undecalcified samples were scanned at a resolution of 9 μm. After obtaining 2D images, 3D image reconstruction was performed.

### 2.11 Histological analysis

After decalcification, the specimens were dehydrated with a gradient of 50% to 100% ethanol, embedded in paraffin, and sectioned on the coronal plane with a thickness of 5 μm. The new bone structure was stained with hematoxylin–eosin (H&E) and Masson's staining and observed under a LeicaDM4000 optical microscope (Leica, Wetzlar, Germany).

### 2.12 Statistical analysis

Quantitative data are expressed as the mean and standard deviation (SD). One-way and two-way analysis of variance (ANOVA), followed by a graph-based test at a significance level of *α* = 0.05, were used to compare multiple sample means. When necessary, Student's *t*-tests were used to analyze data.

## 3. Results and discussion

This study systematically evaluated the performance of the GelMA/HAp/HAM composite membrane in terms of microstructural characteristics, mechanical properties, biocompatibility, osteogenic differentiation, and *in vivo* bone regeneration. The results demonstrate that this bilayer membrane design addresses critical limitations in guided bone regeneration (GBR) applications by combining mechanical stability with enhanced bioactivity.

### 3.1 Microstructure and surface properties

**Fig. 1:**
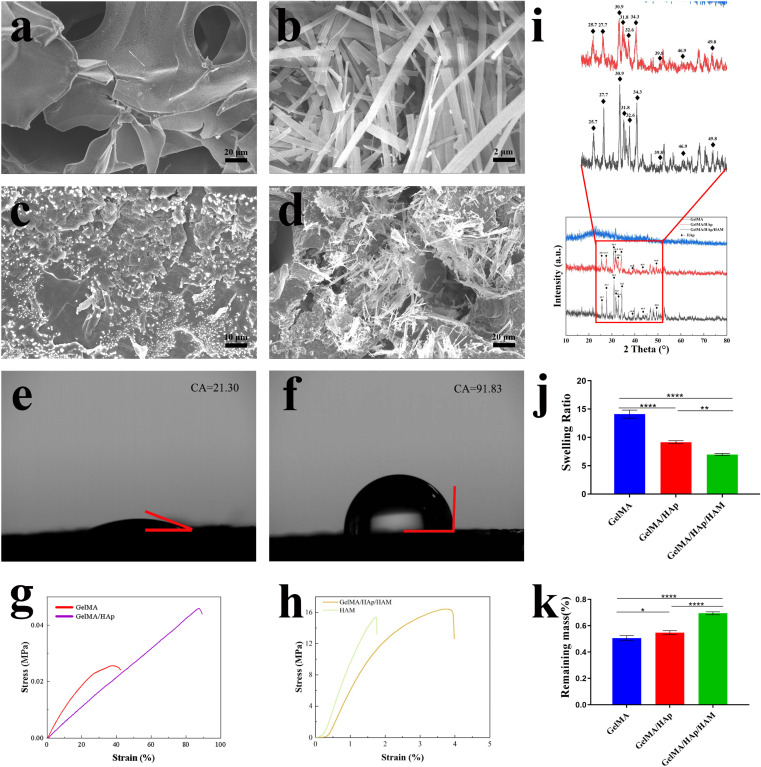
The cross-sectional images of SEM (a) GelMA (scale bar: 20 μm), (b) HAM (scale bar: 2 μm), (c) GelMA/HAp (scale bar: 10 μm), (d) GelMA/HAp/HAM (scale bar: 20 μm), (e) CA of GelMA/HAp, (f) CA of HAM, (g) stress–strain curves of GelMA, GelMA/HAp, (h) stress–strain curves of HAM, GelMA/HAp/HAM, (i) XRD of GelMA, GelMA/HAp, GelMA/HAp/HAM, (j) swelling behavior of GelMA/HAp, GelMA/HAp/HAM, (k) degradation performance of GelMA, GelMA/HAp, GelMA/HAp/HAM,. Data are represented as mean ± SD (*n* = 3). (*P < 0.05, **P < 0.01, ***P < 0.001, ****P < 0.0001.)

SEM images (Fig. 1a–d) revealed the different microstructures of the membrane components: GelMA exhibited a porous structure conducive to nutrient exchange, while HAM exhibited a dense, fibrous architecture ideal for barrier function. The GelMA/HAp layer exhibited a uniform distribution of hydroxyapatite (HAp) particles within its porous network, preserving the interconnected pore structure (50–150 μm) critical for cellular activity. The integration of GelMA/HAp with HAM resulted in a seamless bilayer structure, indicating excellent interfacial stability.

Surface hydrophilicity measurements showed that GelMA/HAp (Fig. 1e) exhibited enhanced hydrophilicity (contact angle: 21.30° ± 0.20°), which, in combination with nanoscale surface protrusions (Fig. 3c), facilitated cell adhesion and proliferation.^31,32^ In contrast, the HAM (Fig. 1f) layer exhibited hydrophobic properties (contact angle: 91.83° ± 0.76°), consistent with its role as a physical barrier to prevent soft tissue invasion.

### 3.2 X-ray diffraction (XRD) analysis

The XRD spectra of GelMA/HAp and GelMA/HAp/HAM showed characteristic peaks for hydroxyapatite (HAp) at 25.7°, 27.7°, 30.9°, 31.8°, 32.6°, 34.3°, 39.8°, 46.9° and 49.8°, confirming the successful incorporation of HAp. Notably, GelMA/HAp/HAM showed significantly higher peak intensities compared to GelMA/HAp, indicating a much higher HAp content in the composite membrane (Fig. 1i).

To quantify this, high temperature calcination experiments revealed that the residual HAp content in GelMA/HAp/HAM was approximately 291.23 ± 4.45 mg compared to only 3.17 ± 0.55 mg in GelMA/HAp, a 91.87-fold increase. This significant increase in HAp content is critical for improving osteoconductivity, as HAp provides sustained release of calcium and phosphate ions to support bone matrix mineralization and osteogenic differentiation.^33–35^

The increased HAp content, combined with the membrane's mechanical stability and bioactive surface, underpins the superior osteogenic performance of GelMA/HAp/HAM observed in both *in vitro* and *in vivo* studies, making it an excellent candidate for guided bone regeneration applications.

### 3.3 Mechanical properties

Mechanical testing (Fig. 1g and h) and (Table 1) highlighted the structural stability of the HAM layer, which exhibited high tensile strength (15.39 ± 2.78 MPa) and elastic modulus (12.99 ± 0.074 MPa). However, its limited elongation at break (1.72 ± 0.13%) compromised its flexibility. Incorporation of GelMA/HAp significantly improved the flexibility of the composite membrane while maintaining mechanical strength. The GelMA/HAp/HAM membrane achieved a tensile strength of 16.43 ± 1.93 MPa and elastic modulus of 9.56 ± 0.068 MPa, with a strain at break (3.77 ± 0.17%) that was 2.19 times higher than that of HAM alone. This combination of strength and flexibility enhances surgical handling and ensures that the membrane can conform to irregular defect geometries without compromising spatial stability, an essential requirement for GBR applications.

**Table 1:** Mechanical properties of different types of membranes, obtained from their respective stress–strain curves (mean 5 SD)

Membranes	Tensile strength(MPa)	Elastic modulus (MPa)	Strain (%)
GelMA	0.026 ± 4.56 × 10^−4^	0.00106 ± 3.78 × 10^−5^	42.4 ± 5.8
GelMA/HAp	0.047 ± 6.38 × 10^−4^	0.00062 ± 8.53 × 10^−6^	89.69 ± 11.3
HAM	15.39 ± 2.78	12.99 ± 0.074	1.72 ± 0.13
GelMA/HAp/HAM	16.43 ± 1.93	9.56 ± 0.068	3.77 ± 0.17

The stress–strain behavior of the GelMA/HAp/HAM membrane closely mimicked that of natural bone, showing a linear increase in stress followed by gradual failure at high strain levels. This suggests the ability of the membrane to withstand physiological loading conditions while maintaining structural integrity.^36,37^

### 3.4 Swelling and degradation characteristics

The swelling test (Fig. 1j) showed that the GelMA/HAp/HAM membrane exhibited a significantly lower swelling ratio (6.97 ± 0.19) compared to GelMA alone (14.12 ± 0.71). This reduction reflects a higher cross-linking density, which improves the structural stability of the membrane.^38^ Similarly, degradation analysis (Fig. 1k) showed a slower degradation rate for the GelMA/HAp/HAM membrane, likely due to the presence of covalent cross-links that provide greater resistance to enzymatic degradation.^39–41^ This controlled degradation profile ensures that the membrane provides sufficient mechanical support during the bone regeneration process, while allowing a gradual transfer of mechanical load to the newly formed tissue.

### 3.5 Biocompatibility and cytotoxicity

**Fig. 2:**
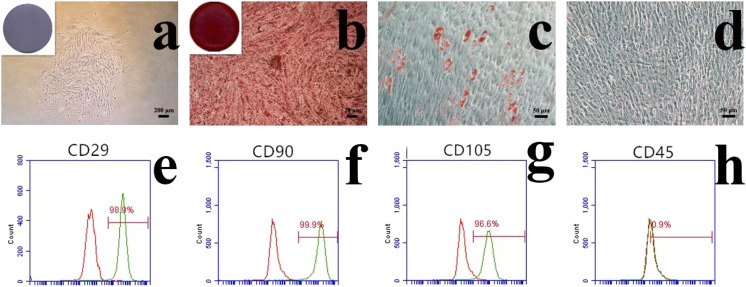
Characteristics of human PDLSCs(hPDLSCs). (a) Single colonies of hPDLSCs after 10 days (scale bar: 200 μm), (b) bone formation experiment with alizarin red staining (scale bar: 20 μm), (c and d) fat formation experiment with oil red O staining, (c) positive, and (d) negative(scale bar: 50 μm), (e–h) flow cytometric analysis demonstrating that hPDLSCs were positive for the mesenchymal stem cells (MSCs) markers (e) CD29, (f) CD90, (g) CD105 and negative for the hematopoietic stem cell (HSCs) marker (h) CD45.

In the current study, cells were isolated from periodontal ligament tissue of healthy periodontal tissue. The isolated cells were amplified *in vitro*. the reproductive potential and multi-lineage differentiation capacity of PDLSCs, a series of assays were conducted. The colony formation assay (Fig. 2a) demonstrated excellent monoclonal formation ability. The osteoblastic differentiation assay, visualized by alizarin red staining (Fig. 2b), showed distinct calcium nodule formation. The adipogenic differentiation assay, evaluated using oil red O staining, exhibited positive results (Fig. 2c), which contrasted with the negative control (Fig. 2d). Flow cytometry analysis (Fig. 2e–h) revealed positive expression of mesenchymal stem cell markers CD29, CD90, and CD105, along with negative expression of the hematopoietic stem cell marker CD45, confirming the mesenchymal stem cell properties of PDLSCs, consistent with previous studies.^42,43^

**Fig. 3:**
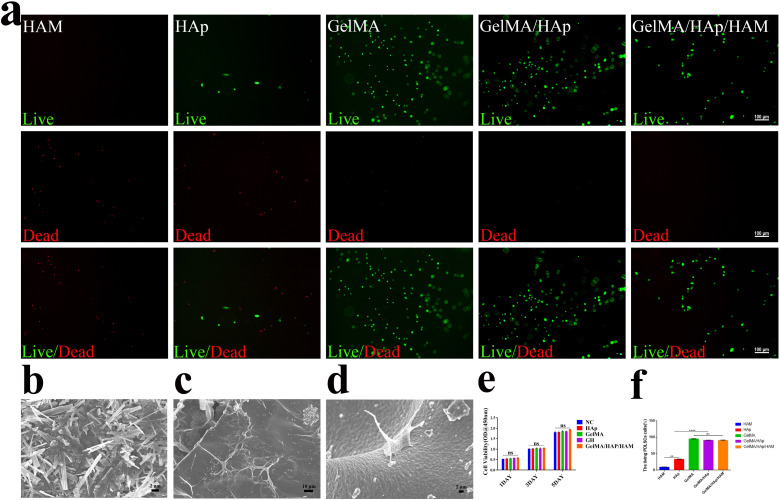
(a). Fluorescence images of live (green)/dead (red) cells and merged images (green and red), SEM images of cells adhesion (scale bar: 100 μm), (b) HAM for barrier layer (scale bar: 2 μm), (c) GelMA/HAp for hydrogel layer (scale bar: 10 μm), (d) PDLSCs adhere on GelMA/HAp (scale bar: 2 μm), (e) cytotoxicity analysis: culture PDLSCs for 1, 3, 5 days in the culture medium after soaking the material to determine the cell proliferation activity. Data are represented as mean ± SD (*n* = 3), (f) Live/dead staining.

Biocompatibility evaluations confirmed that the GelMA/HAp/HAM membrane supported cell adhesion, proliferation, and survival. SEM images of the interface between GelMA/HAp and HAM (Fig. 3b) showed strong bonding without any signs of separation, ensuring excellent structural integrity. Live/dead staining (Fig. 3a and f) showed a high survival rate (>90%) of PDLSCs cultured on the composite membrane, while SEM imaging revealed well-dispersed cells with extended filopodia (Fig. 3d). This enhanced adhesion was attributed to the hydrophilic nature and nanoscale topography of the GelMA/HAp layer (Fig. 3c). Furthermore, the CCK-8 assay (Fig. 3e) showed no significant cytotoxicity, with comparable cell proliferation rates in all experimental and control groups.

### 3.6 Osteogenic differentiation experiment *in vitro*

**Fig. 4:**
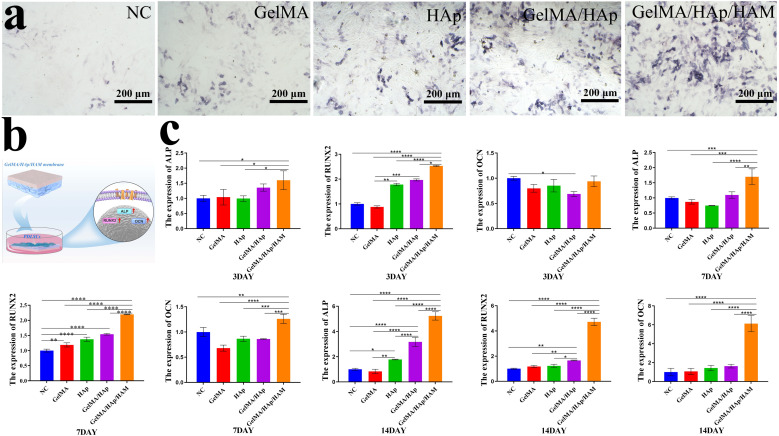
*In vitro* osteogenic differentiation experiments of PDLSCs. (a) Schematic diagram of osteogenic induction culture, (b) ALP staining at 7 days (scale bar: 200 μm), (c) expression of ALP/RUNX2/OCN genes at 3, 7, and 14 days. Data are represented as mean ± SD (*n* = 3). *P < 0.05, **P < 0.01, ***P < 0.001 and ****P < 0.0001.

By 7 days, ALP staining revealed the presence of ALP-positive cells across all experimental groups, with a notably higher number observed in the GelMA/HAp/HAM group compared to the control. This indicates that the GelMA/HAp/HAM group possesses enhanced osteogenic potential (Fig. 4a). We evaluated the effects of the composition and properties of biomaterials on the osteogenic differentiation potential. Membranes were placed into 6-well plates and cultured in osteogenic medium containing dexamethasone, l-ascorbic acid-2-phosphate, and phosphate. After 3, 7, and 14 days of osteogenic differentiation, the molecular mechanisms were analyzed through gene expression studies focusing on ALP, Runx2, OCN (Fig. 4b and c).

Our RT-PCR analysis revealed distinct differences across the three time points (Table 2). At 3 days, the expression of ALP in the GelMA/HAp/HAM group was approximately 1.6 times higher than the control group, which was statistically significant (*P* < 0.05). By 7 days, ALP expression continued to rise, reaching 1.7 times that of the control group (*P* < 0.001). At 14 days, ALP expression in all groups increased significantly, with the GelMA/HAp/HAM group exhibiting the highest expression level, at 5.24 times that of the control group (*P* < 0.0001). Similarly, Runx2 expression in the GelMA/HAp/HAM group showed a progressive increase over time, with the most pronounced difference observed on 14 days, where its expression was 6.12 times higher than the control group (*P* < 0.0001). In contrast, OCN expression did not significantly differ in 3 days. However, by 7 and 14 days, the GelMA/HAp/HAM group exhibited a substantial increase in OCN expression, with the highest level observed at 14 days, 4.71 times that of the control group (*P* < 0.0001). These results suggest that the GelMA/HAp/HAM barrier membrane significantly promotes osteogenic differentiation over time, likely due to the slow and sustained release of calcium ions from HAp within the membrane.^33,34^ Additionally, the data indicate that HAp possesses excellent bone conductivity, facilitating bone calcification.

**Table 2:** mRNA primer sequences

	5′-3′ Forward primer	5′-3′ Reverse primer
GAPDH	GCACCGTCAAGGCTGAGAAC	TGGTGAAGACGCCAGTGGA
ALP	ATGGGATGGGTGTCTCCACA	CCACGAAGGGGAACTTGTC
RUNX2	TCCACACCATTAGGGACCATC	TGCTAATGCTTCGTGTTTCCA
OCN	TCACACTCCTCGCCCTATT	GATGTGGTCAGCCAACTCG

### 3.7 *In vivo* bone regeneration

**Fig. 5:**
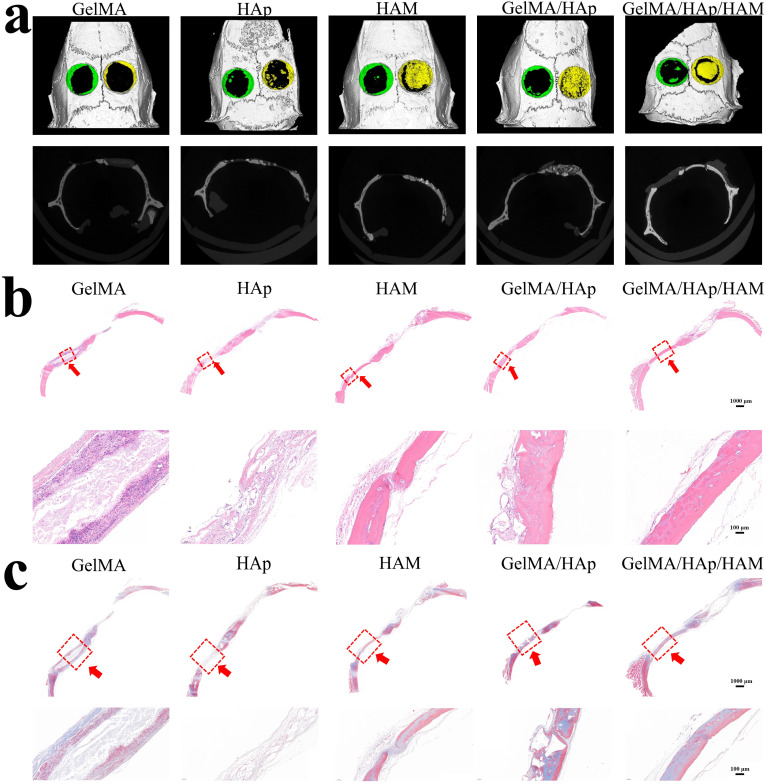
Radiographical analysis of bone formation. (a) Micro-CT images of bone defects of blank group, covered with GelMA, HAp, HAM, GelMA/HAp, and GelMA/HAp/HAM after 8 weeks post-surgery. (b) HE staining is used for the regeneration of bone tissue from cross-section at 8 weeks (scale bar: 1000 μm, 100 μm). (c) Masson staining for regeneration bone tissue of cross-section at 8 weeks (scale bar: 1000 μm, 100 μm).

The superior bone regeneration performance of the GelMA/HAp/HAM membrane was confirmed by micro-CT imaging and histological analysis in a rat calvarial defect model. After eight weeks, the GelMA/HAp/HAM group exhibited a significant degree of new bone formation in comparison to the other groups (Fig. 5a). Histological staining further demonstrated the presence of well-organized bone tissue, with hematoxylin–eosin (H & E) and Masson staining (Fig. 5b and c) corroborating the micro-CT findings. The HAM layer effectively prevented soft tissue infiltration, while the GelMA/HAp layer provided osteoconductivity, promoting new bone growth.^44^

The GelMA/HAp/HAM composite membrane represents a significant advancement in GBR technology, offering a multifunctional platform that combines mechanical stability, controlled degradation, and osteogenic bioactivity. The HAM layer provides spatial stability and acts as a physical barrier, while the GelMA/HAp layer delivers a hydrophilic, osteoconductive surface, thereby enhancing cell adhesion, proliferation, and differentiation. The membrane's high tensile strength, improved flexibility, and controlled degradation profile render it well-suited for surgical applications, particularly in complex defect geometries. *In vitro* and *in vivo* results demonstrate its superior osteogenic potential and biocompatibility.

## 4. Conclusion

We have developed an GelMA/HAp/HAM composite barrier membrane as an advanced strategy for bone defect regeneration. Its bilayer structure endows it with dual functions: acting as a barrier and promoting osteogenesis. The dense layer of HAM serves as a shield to prevent the invasion of connective tissue, while the porous structure of the GelMA/HAp hydrogel facilitates bone regeneration. This innovative composite membrane presents a promising advancement in biomaterial strategies for bone defect repair, offering valuable insights for the future design of therapeutic membranes.

## Data availability

The data will be available from the corresponding author upon reasonable request.

## Author contributions

Qingbin Han: conceptualization, methodology, material synthesis, *in vivo* and *in vitro* experiments, analysis, writing. Delu Zhao: material synthesis, analysis, writing. Xiaohong Wang: analysis, writing, editing. Mengyao Shang: analysis, reviewing and editing. Wenbin Zhou: material synthesis, reviewing. Qing Li: supervision, resources, conceptualization, reviewing. Hui Song: supervision, resources, conceptualization, reviewing.

## Conflicts of interest

There are no conflicts to declare.

## Supplementary Material

RA-015-D4RA07623K-s001
