# Supplementary material for: Composite barrier membrane for bone regeneration: advancing biomaterial strategies in defect repair
Source: RSC Adv. 2025 Jan 15;15(2):1290–9. doi: 10.1039/d4ra07623k (PMC11733738; doi:10.1039/d4ra07623k)
Supplement: RA-015-D4RA07623K-s001 [file RA-015-D4RA07623K-s001.pdf]

Supplementary Information (SI) for RSC Advances.
This journal is © The Royal Society of Chemistry 2025

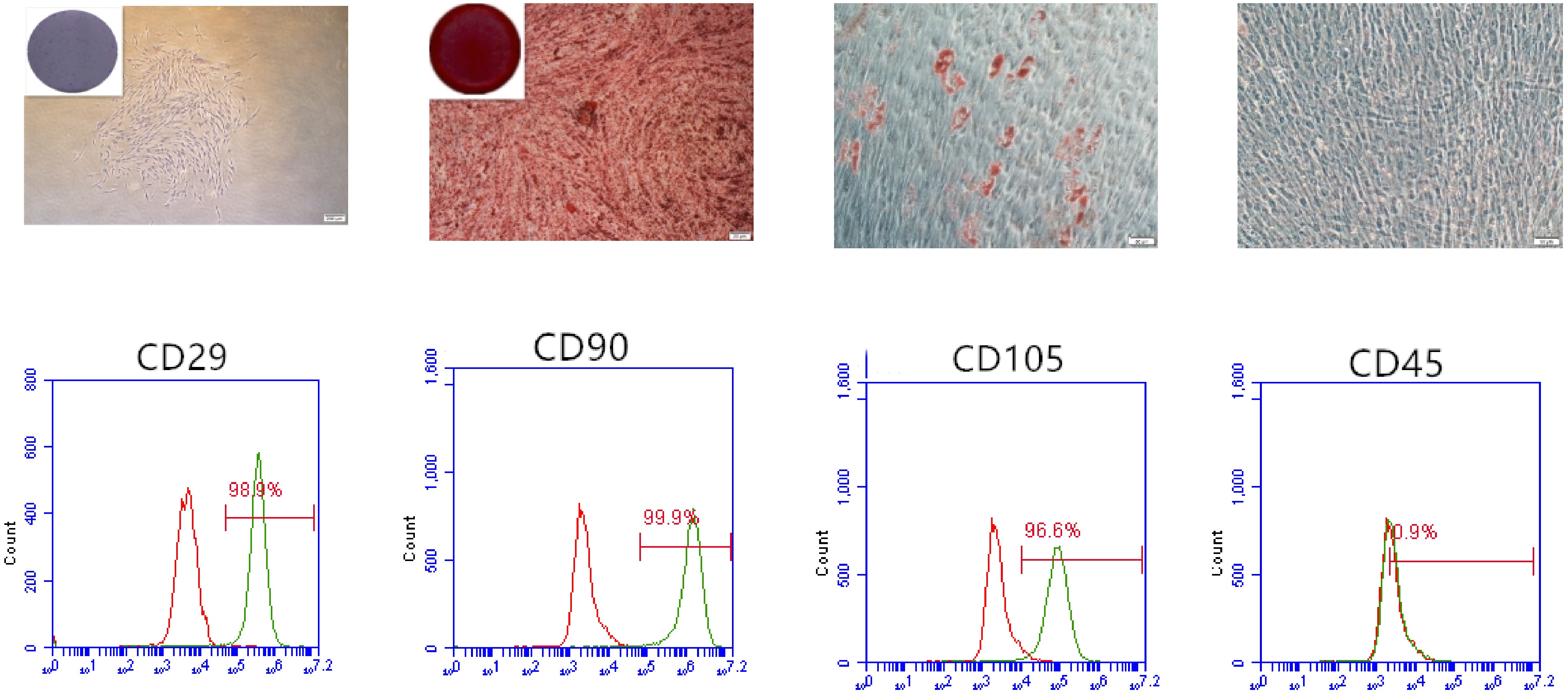

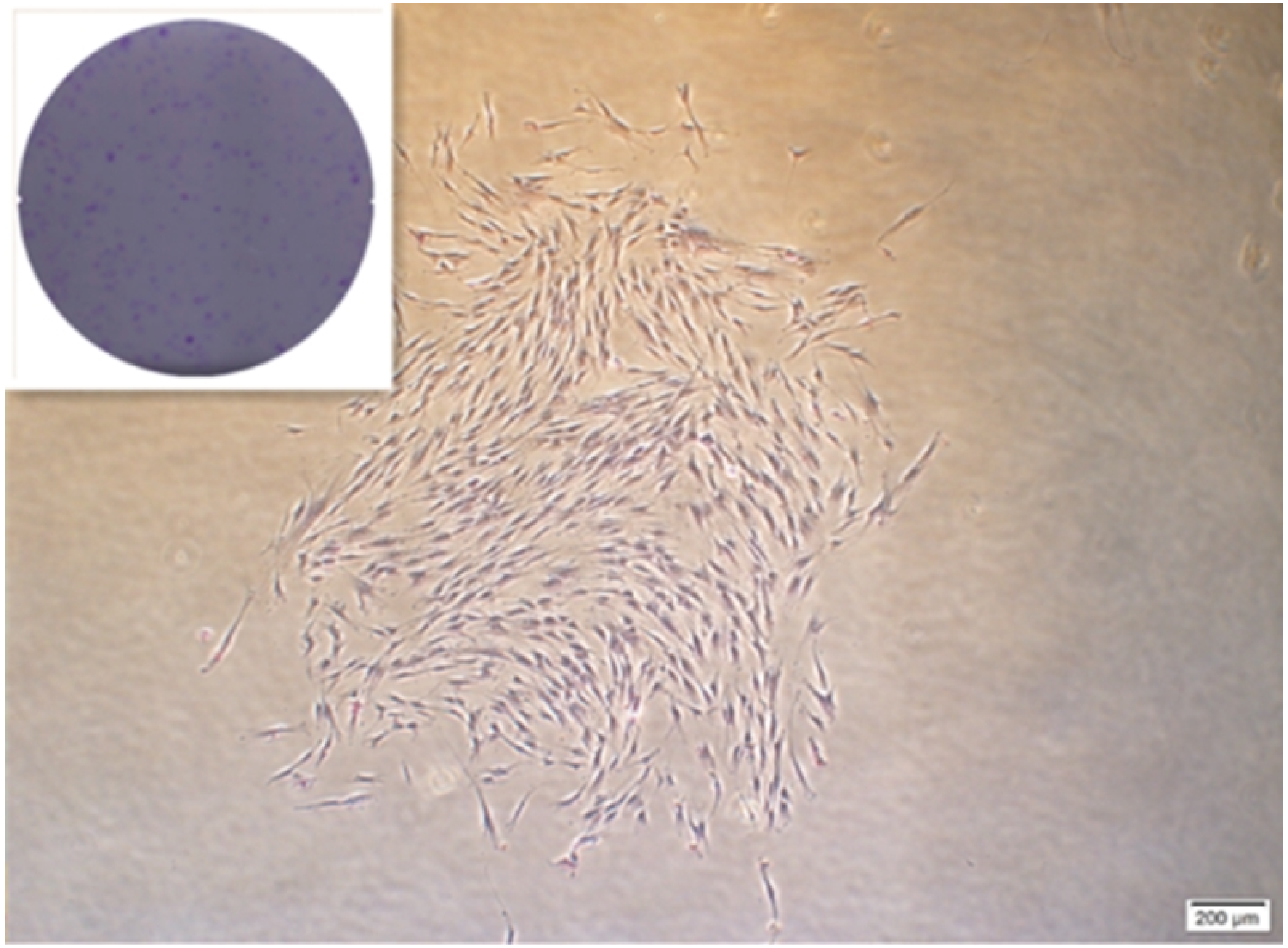

a. Single colonies of hPDLSCs after 10 d.

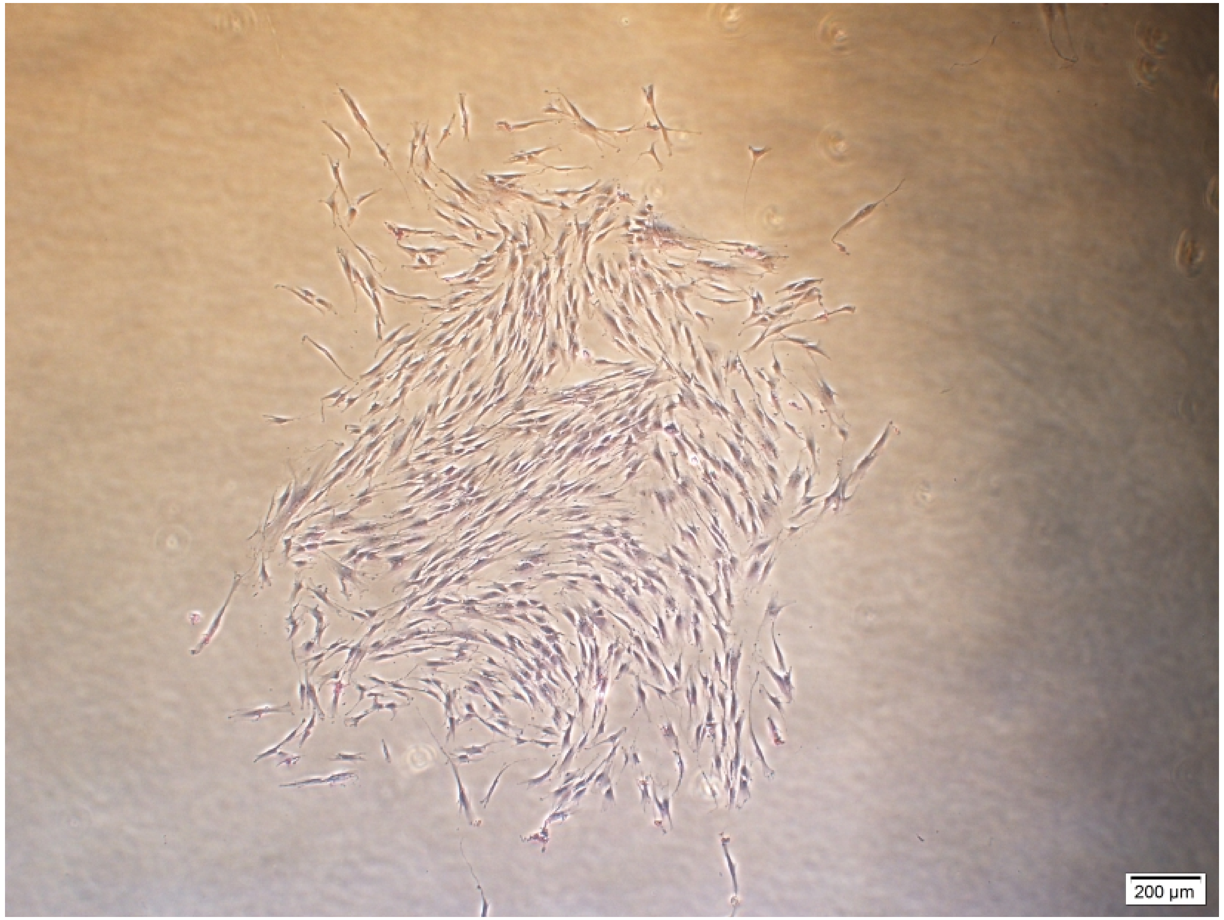

a. Single colonies of hPDLSCs after 10 d.

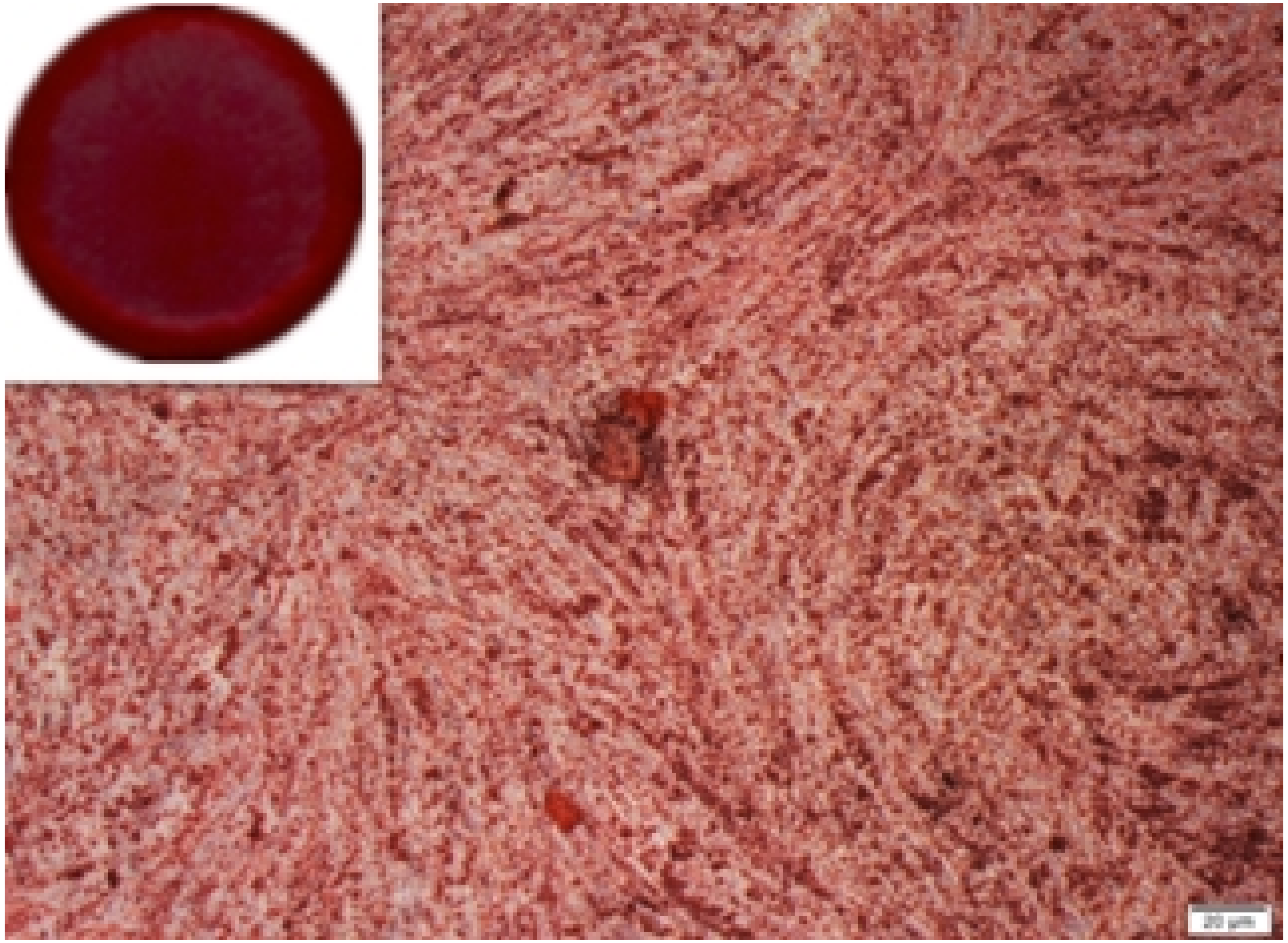

b. Bone formation experiment with alizarin red staining.

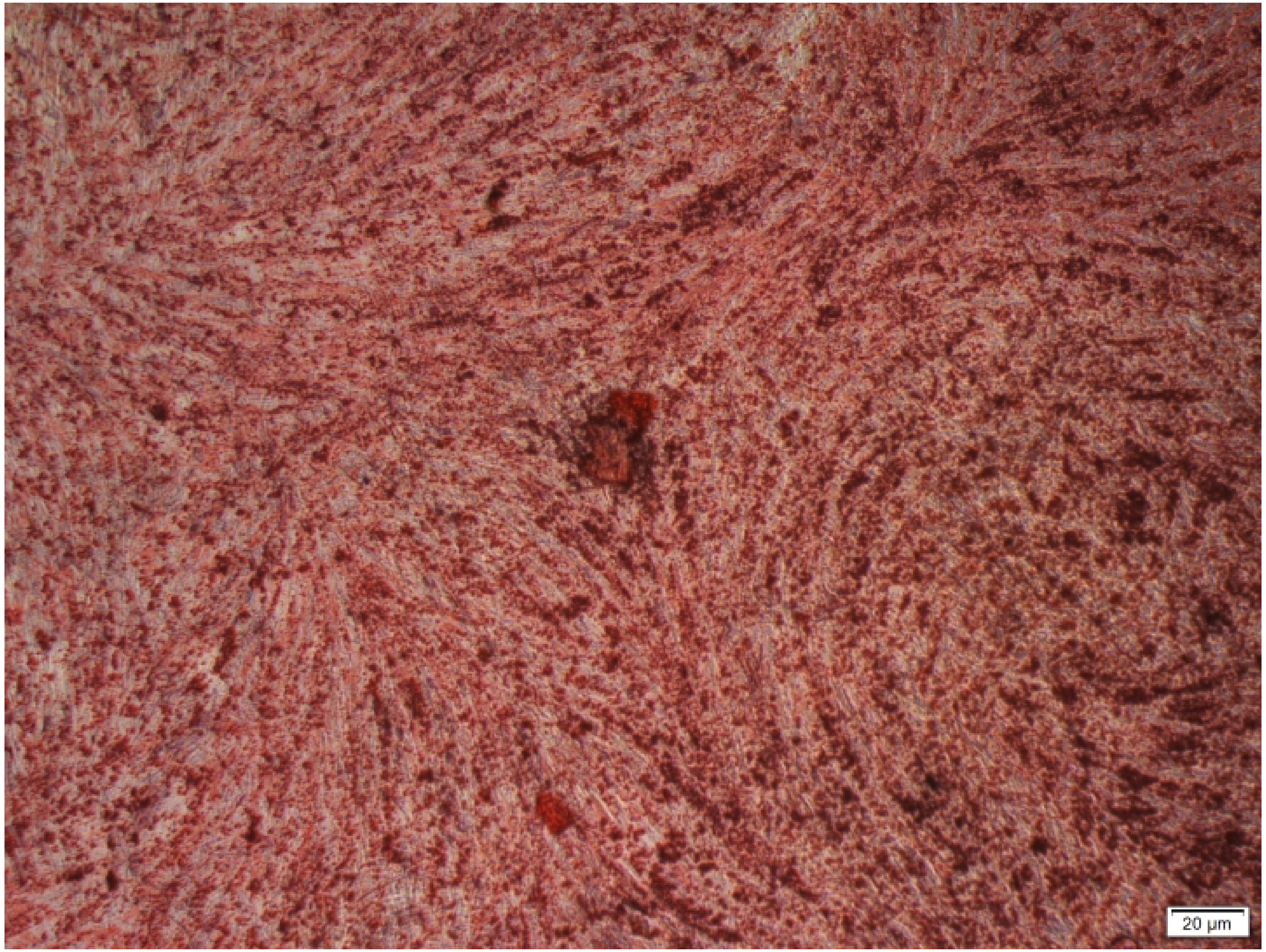

b. Bone formation experiment with alizarin red staining.

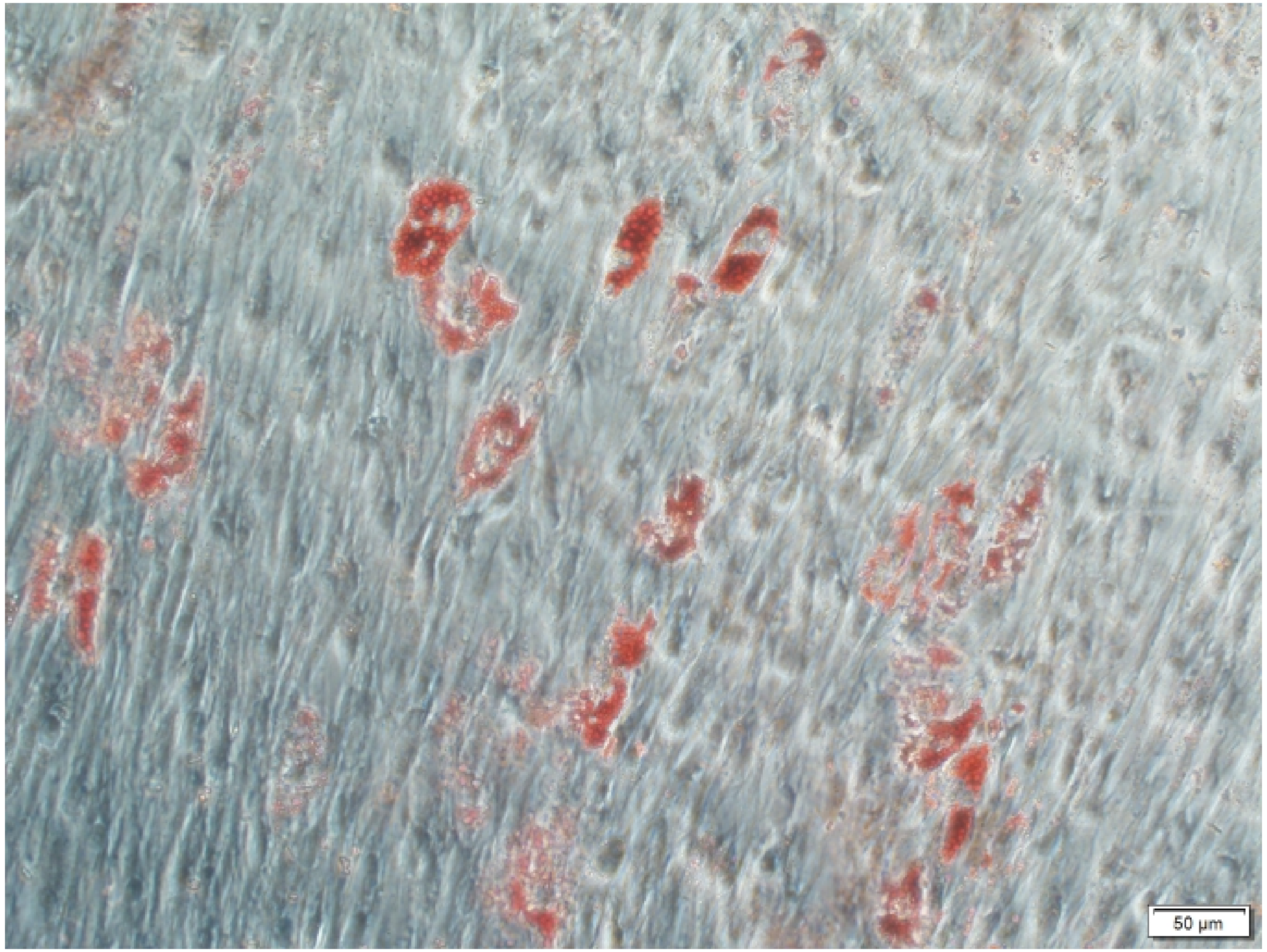

c. Positive fat formation experiment with oil red O staining

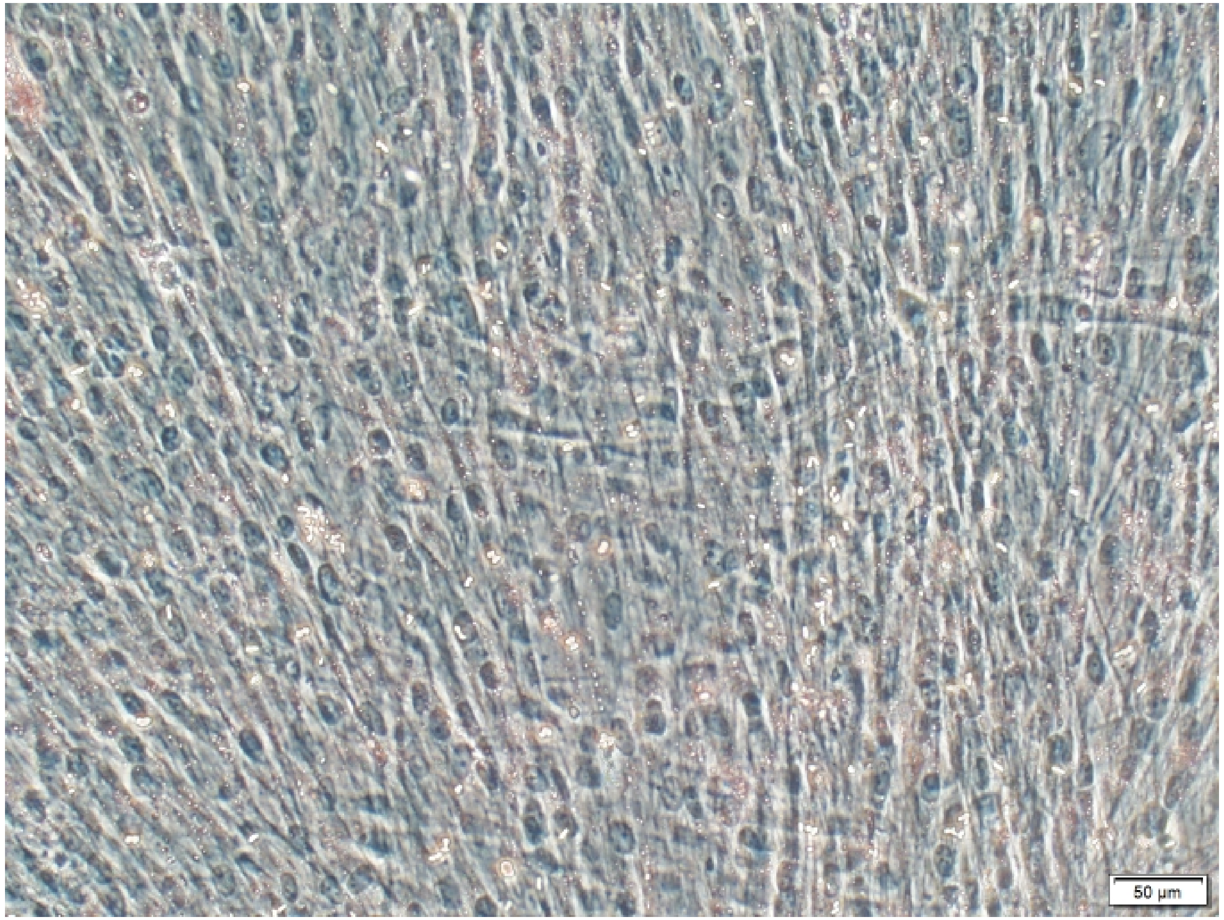

d. Negative fat formation experiment with oil red O staining

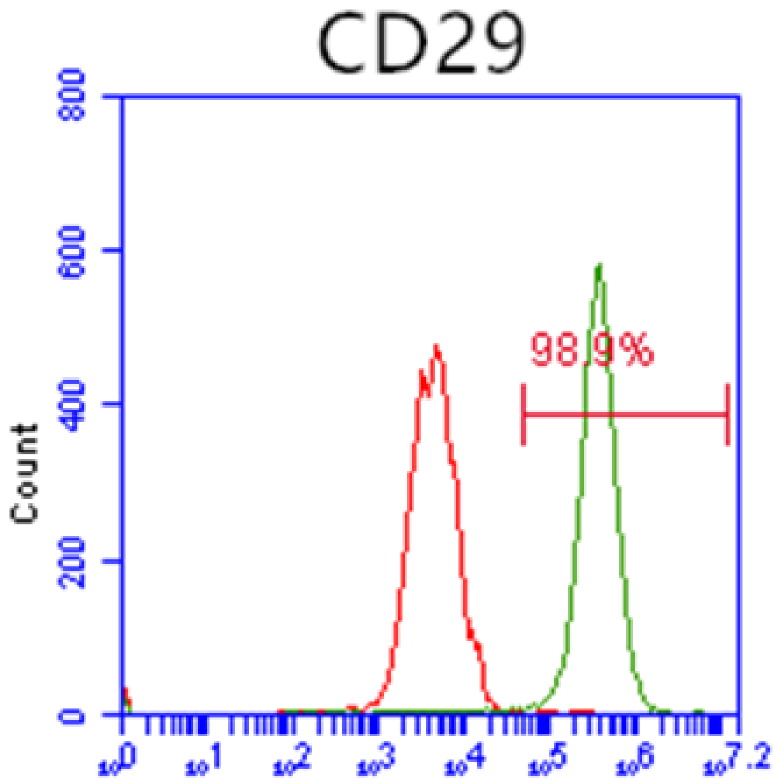

e. Positive for the mesenchymal stem cells (MSCs) markers CD29.

.

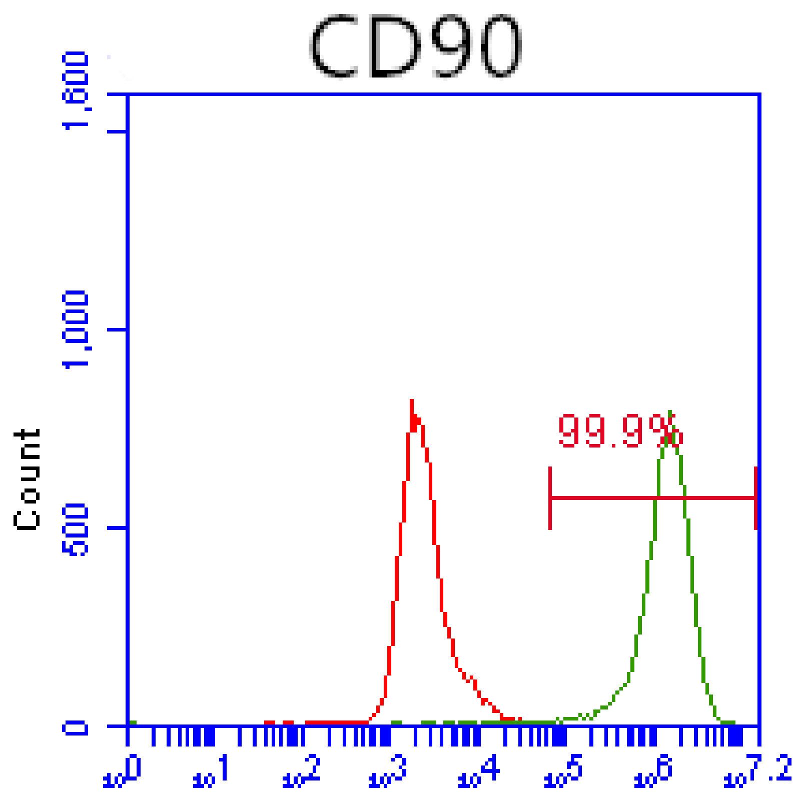

f. Positive for the mesenchymal stem cells (MSCs) markers CD90.

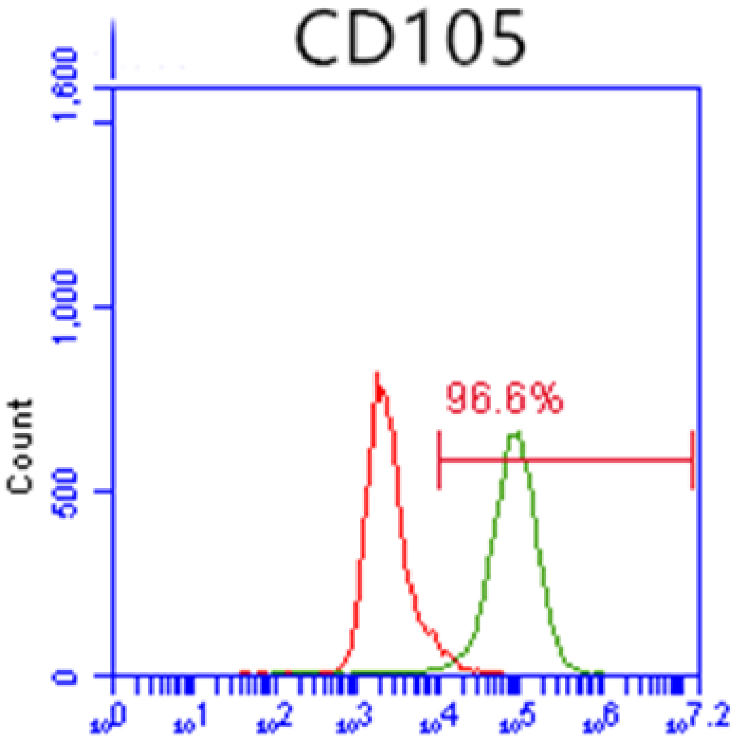

g. Positive for the mesenchymal stem cells (MSCs) markers CD105.

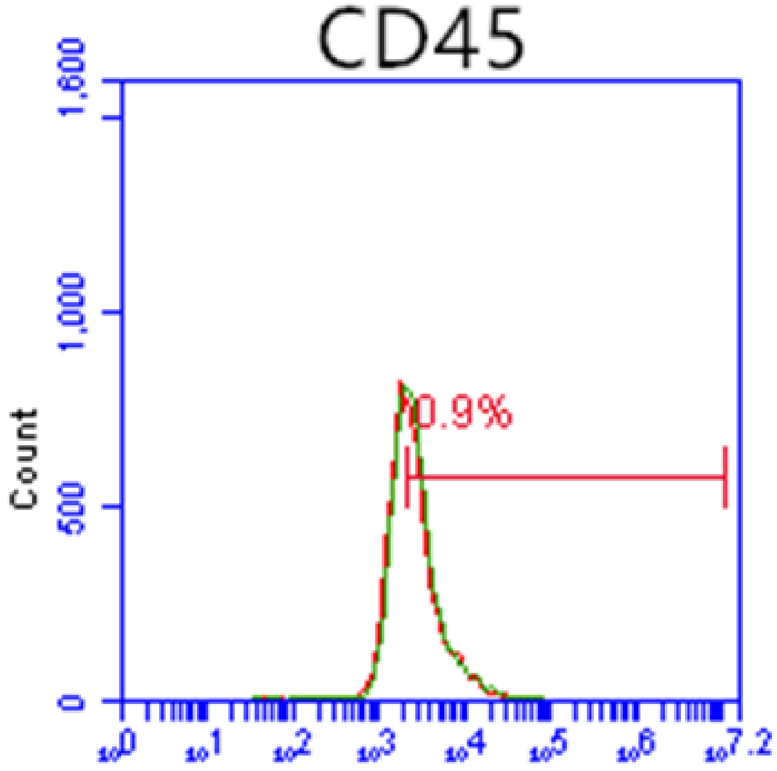

h. negative for the mesenchymal stem cells (MSCs) markers CD105.
